# AEROBIC EXERCISE ATTENUATES HEPATIC LIPID PEROXIDATION IN AN EXPERIMENTAL MODEL OF OBESITY-ASSOCIATED NAFLD

**DOI:** 10.1590/S0004-2803.24612025-086

**Published:** 2026-05-18

**Authors:** Maria Luiza Rodrigues Pereira LIMA, Carolina Rosa GIODA, Laura Hora Rios LEITE, Cândido Celso COIMBRA, Bernardo Henrique Mendes CORREA, Virginia Hora Rios LEITE, Teresa Cristina Abreu FERRARI

**Affiliations:** 1 Universidade Federal de Minas Gerais, Hospital das Clínicas, Instituto Alfa de Gastroenterologia, Belo Horizonte, MG, Brasil.; 2 Universidade Federal de Minas Gerais, Faculdade de Medicina, Departamento de Anatomia Patológica e Medicina Legal, Belo Horizonte, MG, Brasil.; 3 Universidade Federal de Minas Gerais, Instituto de Ciências Biológicas, Departamento de Bioquímica e Imunologia, Belo Horizonte, MG, Brasil.; 4 Universidade Federal de Juiz de Fora, Instituto de Ciências Biológicas, Departamento de Biofísica e Fisiologia, Juiz de Fora, MG, Brasil.; 5 Universidade Federal de Minas Gerais, Instituto de Ciências Biológicas, Departamento de Fisiologia e Biofísica, Belo Horizonte, MG, Brasil.; 6 Universidade Federal de Minas Gerais, Faculdade de Medicina, Belo Horizonte, MG, Brasil.; 7 Universidade Federal de Minas Gerais, Faculdade de Medicina, Departamento de Clínica Médica, Belo Horizonte, MG, Brasil.

**Keywords:** Malondialdehyde, non-alcoholic fatty liver disease, oxidative stress, Malondialdeido, doença hepática gordurosa não alcoólica, estresse oxidativo

## Abstract

**Background:**

The global rise in obesity has been accompanied by an increasing prevalence of nonalcoholic fatty liver disease (NAFLD), for which effective non-pharmacological therapeutic strategies remain limited.

**Objective:**

This study investigated the effects of aerobic exercise on hepatic oxidative stress in an experimental model of obesity-associated NAFLD.

**Methods:**

Newly weaned Wistar rats were fed a highly palatable, obesity-inducing diet. After obesity was established, the animals were randomly assigned to either a trained group (n=12) or a sedentary group (n=12). The trained group underwent moderate-intensity treadmill running for eight weeks. Hepatic lipid peroxidation was assessed using the TBARS (thiobarbituric acid reactive substances) assay.

**Results:**

Aerobic training significantly reduced hepatic TBARS levels (*P*<0.0005), in an average of 1.8 nmol MDA/mg protein compared to the sedentary group. These benefits were significant regardless of weight gain maintenance.

**Conclusion:**

The findings suggest that regular physical exercise attenuates hepatic lipid peroxidation in an experimental model of obesity-associated NAFLD. The results support that physical exercise is an effective non-pharmacological strategy for modulating oxidative stress and preventing disease progression.

## HIGHLIGHTS

• Aerobic exercise effect on hepatic oxidative stress in a model of obesity-associated NAFLD was assessed.

• Hepatic TBARS analysis was conducted in obese-induced and exercise-trained rats.

• Hepatic lipid peroxidation decreases in obesity-associated NAFLD after exercise training.

• Exercise mitigates liver damage in NAFLD, independent of body weight change.

## RESUMO

**Contexto:**

O aumento global da obesidade tem sido acompanhado de prevalência crescente da doença hepática gordurosa não alcoólica (DHGNA), para a qual ainda são limitadas estratégias terapêuticas não farmacológicas eficazes.

**Objetivo:**

Este estudo investigou os efeitos do exercício aeróbico sobre o estresse oxidativo hepático em um modelo experimental de obesidade associada à DHGNA.

**Métodos:**

Ratos Wistar recém-desmamados foram alimentados com dieta altamente palatável e indutora de obesidade. Após o estabelecimento da obesidade, os animais foram divididos aleatoriamente em grupos treinados (n=12) e sedentários (n=12). O grupo treinado foi submetido à corrida em esteira de intensidade moderada por oito semanas. A peroxidação lipídica hepática foi avaliada por meio do método TBARS (substâncias reativas ao ácido tiobarbitúrico).

**Resultados:**

O treinamento aeróbico reduziu significativamente os níveis hepáticos de TBARS (*P*<0,0005), em uma média de 1,8 nmol MDA/mg de proteína em comparação ao grupo sedentário. Esses benefícios foram evidentes, apesar da manutenção do ganho de peso.

**Conclusão:**

Os achados sugerem que o exercício físico regular atenua a peroxidação lipídica hepática em modelo experimental de DHGNA associada à obesidade. Os resultados indicam que o exercício físico é uma estratégia não farmacológica eficiente na modulação do estresse oxidativo e na prevenção da progressão da doença.

## INTRODUCTION

The global rise in obesity has been paralleled by an increasing prevalence of non-alcoholic fatty liver disease (NAFLD). It is estimated that NAFLD affects between 60% and 95% of individuals with obesity, and 5% to 25% of the general population^1^. Furthermore, a Brazilian study conducted by Lima et al. reported that 99.1% of patients with morbid obesity undergoing bariatric surgery exhibited various forms of NAFLD, with more than half presenting with advanced stages of the disease^2^.

NAFLD is a multifactorial condition characterized by complex interactions among genetic predisposition, age, sex, metabolic disorders, and lifestyle factors, resulting in distinct clinical phenotypes. The disease spectrum ranges from simple hepatic steatosis to non-alcoholic steatohepatitis (NASH), fibrosis, cirrhosis, and hepatocellular carcinoma. Among the mechanisms implicated, lipid peroxidation and oxidative stress play a central role in the progression from steatosis to NASH^3^.

Although previous studies have shown that regular aerobic exercise can attenuate hepatic oxidative stress and reduce lipid peroxidation, these effects have been reported in heterogeneous experimental models, not consistently representative of obesity-associated NAFLD induced by a high-sucrose diet^4^. In the present study, we employed an experimental model of obesity-associated NAFLD induced by sucrose-rich feeding in Wistar rats, aiming to assess the effects of aerobic exercise on hepatic lipid peroxidation.

## METHODS

This is an experimental study approved by the Ethics Committee on Animal Experimentation of the Universidade Federal de Minas Gerais, Belo Horizonte, Brazil for Care and Use of Laboratory Animals (CETEA 53/2007) and was carried out in accordance with the regulations described in the Committee’s Guiding Principles Manual.

### Animals

The study included an experimental group (EG) and a control group (CG), each consisting of 12 newly weaned male Wistar rats. They were kept in individual cages in a hygienic environment with controlled temperature, humidity, and light, and had free access to water and food. After being weighed and measured, the animals were randomly assigned to either the EG or CG group.

### Diet and physical training

Animals in the CG received standard chow (NUVILAB-CR1), providing 309 kcal per 100 g of dry matter, with 24.8 g protein, 3.4 g lipids, 44.8 g carbohydrates, 8.2 g minerals, and 18.8 g dietary fiber. The EG received a hypercaloric, palatable diet composed of powdered chow (33%), condensed milk (33%), refined sugar (7%), and water (27%), yielding 339 kcal per 100 g of dry matter, with 16.1 g protein, 3.4 g lipids, 61.0 g carbohydrates, 5.1 g minerals, and 14.4 g fiber. Diets were freshly prepared and offered daily in weighed portions. Food intake was monitored by weighing the residual feed^5^.

EG animals underwent physical training consisting of moderate-intensity treadmill running every day, 5 days a week, for 8 weeks. The speed and duration were gradually increased from 10m/min to 25 m/min at a 5% incline for 60 minutes^6^. Weekly anthropometric measurements were recorded^7^.

### TBARS analysis

After 30 weeks, the rats were euthanized, and liver samples were collected for TBARS analysis using the Ohkawa method^8^. Results were expressed as nmol MDA/mg protein.

### Statistical analysis

Data were described as mean ± standard deviation or median and interquartile range, depending on the distribution, as determined by the Shapiro-Wilk test. Comparisons were performed using unpaired t-tests or Mann-Whitney U tests. Statistical significance was set at *P*<0.05 using IBM SPSS Statistics version 26.0.

## RESULTS

The comparative results of the anthropometric characteristics and TBARS levels between the two groups are described in Table 1.

**TABLE 1:** Anthropometric data and TBARS levels in animals submitted or not to physical training.

Characteristics*	Physical training		*P*
	Yes (n=12)	No (n=12)	Value
Initial weight (g)	96.3±19.4	100.7±15.8	0.550 ^1^
Final weight (g)	559,3[87,2]	621.5 [191,1]	0.840 ^2^
Δ BMI (g/cm^2^)	0.4 [0.3-0.5]	0.5 (0.3-0.6)	0.126 ^2^
TBARS (nmol MDA/mg protein)	6.3±1.0	8.1±0.6	0.005 ^1^

*Values are expressed as the mean value ± standard deviation, or median (IQR). BMI: body mass index. TBARS: thiobarbituric acid reactive substances. ^1^t-student test. ^2^Mann-Whitney test.

**FIGURE 1:**
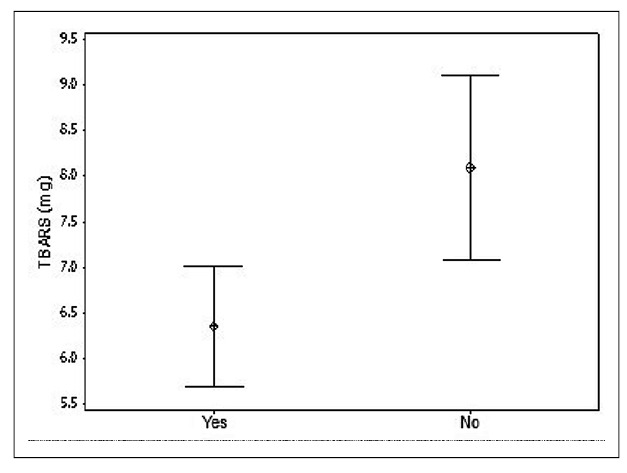
Mean TBARS (mg) of rats submitted to (yes) and not submitted to (no) physical training.

Rats that underwent physical training had, on average, 1.8 nmol MDA/mg protein less TBARS than those that did not exercise regularly (Figure 1).

## DISCUSSION

The present study shows that moderate-intensity aerobic exercise significantly reduces hepatic lipid peroxidation in an experimental model of obesity-associated NAFLD. Trained animals showed a reduction in hepatic TBARS levels compared to sedentary controls, despite unchanged body weight gain. These data reinforce that regular physical activity mitigates oxidative stress, regardless of changes in body weight.

The validity of our experimental model is supported by prior research using this sucrose-rich diet to induce obesity-associated NAFLD in Wistar rats^5^. Our findings are consistent with previous reports indicating that aerobic exercise enhances hepatic mitochondrial function and antioxidant defenses in models of diet-induced obesity^9^ ^,^ ^10^. The study design incorporated a validated animal model, proper randomization, controlled environmental conditions, and rigorous statistical analysis, increasing the credibility and reproducibility of our results.

Although TBARS is recognized as a nonspecific method for malondialdehyde detection, it is widely used as a surrogate marker of lipid peroxidation in experimental research, facilitating valid comparisons among studies^11^ ^,^ ^12^. Future investigations should consider more specific markers, such as 4-hydroxynonenal or protein carbonyls, to further refine the assessment of oxidative damage.

The probable mechanism underlying physical exercise-mediated hepatoprotection includes a reduction in intrahepatic fat content, suppression of ROS overproduction, enhancement of fatty acid β-oxidation, and preservation of hepatic autophagy^3^ ^,^ ^10^. Additionally, the decreased lipid peroxidation induced by physical exercise limits mitochondrial dysfunction and the activation of pro-inflammatory and pro-fibrotic pathways, reducing hepatocyte apoptosis and fibrogenesis^13^ ^,^ ^14^.

Taken together, the current evidence reinforces the role of aerobic exercise as an effective non-pharmacological intervention for reducing hepatic oxidative stress and preventing NAFLD progression in obesity-associated conditions. The reproducibility and statistical robustness of our findings contribute to the growing body of evidence supporting exercise prescription as a therapeutic strategy.

## CONCLUSIONS

Aerobic exercise is effective in reducing lipid peroxidation, as assessed by TBARS levels in the liver tissue of obesity-associated NAFLD rats. These findings reinforce the role of regular physical activity as a non-pharmacological therapeutic intervention for NAFLD, even in the absence of weight loss.

## Data Availability

Data-available-upon-request
